# DRAM Triggers Lysosomal Membrane Permeabilization and Cell Death in CD4^+^ T Cells Infected with HIV

**DOI:** 10.1371/journal.ppat.1003328

**Published:** 2013-05-02

**Authors:** Mireille Laforge, Sophie Limou, Francis Harper, Nicoletta Casartelli, Vasco Rodrigues, Ricardo Silvestre, Houda Haloui, Jean-Francois Zagury, Anna Senik, Jerome Estaquier

**Affiliations:** 1 CNRS FRE 3235, Université Paris Descartes, Paris, France; 2 Chaire de Bioinformatique, Conservatoire National des Arts et Métiers, Paris, France; 3 FRE 2937-Génétique Moléculaire et Intégration des Fonctions Cellulaires, Villejuif, France; 4 Institut Pasteur, Unité Virus et Immunité, Paris, France; 5 Instituto de Biologia Molecular e Celular, Universidade do Porto, Porto, Portugal; 6 Université Laval, Centre de Recherche du CHU de Québec, Québec, Canada; Vanderbilt University School of Medicine, United States of America

## Abstract

Productive HIV infection of CD4^+^ T cells leads to a caspase-independent cell death pathway associated with lysosomal membrane permeabilization (LMP) and cathepsin release, resulting in mitochondrial outer membrane permeabilization (MOMP). Herein, we demonstrate that HIV infection induces damage-regulated autophagy modulator (DRAM) expression in a p53-dependent manner. Knocking down the expression of DRAM and p53 genes with specific siRNAs inhibited autophagy and LMP. However, inhibition of Atg5 and Beclin genes that prevents autophagy had a minor effect on LMP and cell death. The knock down of DRAM gene inhibited cytochrome C release, MOMP and cell death. However, knocking down DRAM, we increased viral infection and production. Our study shows for the first time the involvement of DRAM in host-pathogen interactions, which may represent a mechanism of defense via the elimination of infected cells.

## Author Summary

Lysosomes are acidic organelles capable of digesting macromolecules and regulating autophagy. In the context of host-pathogen interactions, productive viral infections are associated with lysosome membrane permeabilization (LMP) and programmed cell death (PCD). At a molecular level, the tumor suppressor protein 53 (p53), which is a key player in the detection of DNA damage, acts also as a sensor of pathogen replication. Activation of p53 has been considered to be an altruistic cell suicide mechanism that limits viral infection. Here, we provide new evidence that damage-regulated autophagy modulator (DRAM), a p53 target gene, regulates both LMP and PCD of HIV-infected CD4 T cells. Whereas the inhibition of DRAM or p53 prevents autophagy in infected cells, the inhibition of the autophagy machinery has a minor role in this context. As a consequence, the silencing of DRAM leads to increased HIV viral infection. This is the first report describing the role of DRAM in the context of host-pathogen interaction. Whereas it is to the advantage of the pathogens to preserve their hosts and thus facilitate their multiplication and dissemination, hosts have developed altruistic cellular processes to defend themself and limit the spread of the infectious agent in multicellular organisms. We propose that the ancestral DRAM protein represents a mechanism of self-defense, inducing elimination of infected cells through LMP.

## Introduction

Several proteolytic processes are involved in programmed cell death. Lysosomal membrane permeabilization (LMP) and mitochondrial outer membrane permeabilization (MOMP) have been identified as major events triggering programmed cell death. Thus, in a number of models, lysosomal destabilization plays an early and important role in cell death [1], [2]. Lysosomes are acidic organelles that contain numerous acid hydrolases capable of digesting macromolecules of the cell. Upon LMP, the cathepsins are released to the cytosol, where they can initiate the intrinsic apoptotic pathway. This process is mediated in part by the proteolytic activation of the pro-apoptotic molecules, Bid and Bax, resulting in MOMP and cytochrome C release [3]. Thereafter, the release of cytochrome C causes the activation of effector caspases and triggers a caspase-dependent apoptotic pathway. However, lysosome leakage can also induce a caspase-independent non-apoptotic cell death pathway [4]. Thus, lysosomal hydrolases and proteases are acting as initiators and effectors of programmed cell death. CD4^+^ T cells productively infected with HIV-1 die through a caspase-independent death pathway [5], [6], [7], [8], [9]. Treatment of productively infected CD4^+^ T cells with the reverse transcriptase inhibitor DDI prevents programmed cell death [5], [7], [10]. The death of HIV-infected CD4^+^ T cells is associated with the limited permeabilization of lysosomes and lysosomal efflux of cathepsins to the cytosol [10]. Cathepsin D induces conformational change of Bax and its insertion into the OMM promotes the release of cytochrome C [10]. LMP is induced by both the X4 and R5 laboratory strains and by HIV-1 isolates from infected patients. Thus, the permeabilization of lysosomes precedes that of mitochondria and represents an early commitment to cell death in HIV-infected CD4^+^ T cells [10].

Activation of the tumor suppressor p53 can trigger a primary lysosomal destabilization [11], [12]. Induction of its proapoptotic target genes in virally infected cells has been considered as an altruistic suicide mechanism that limits viral infection. Thus, many viruses, including simian virus 40 (SV40), human papilloma virus (HPV) and adenoviruses (Ad), have evolved mechanisms to prevent p53 responses [13], whereas active p53 was detected with several other types of viruses, such as vesicular stomatitis virus (VSV), Newcastle disease virus (NDV) [14] and human immunodeficiency virus (HIV) [15]. However, the mechanism by which p53 mediates LMP is so far unknown.

Lysosomes are also important by their ability to regulate the terminal steps of autophagy [16], [17], [18]. Autophagy is an evolutionarily conserved process first defined genetically in yeast [19], [20]. The primary function of autophagy in most cell types is thought to be an adaptive response to starvation, and is essential for cell survival by degrading proteins and organelles damaged during oxidative stress. In some cellular settings, it can serve as a cell death pathway by itself [21] or in collaboration with apoptosis [22], although its role in this regard is still debated. The cross-talk between apoptosis and autophagy is therefore quite complex, and sometimes contradictory. It has been suggested that autophagy is involved in HIV viral replication [23], in the death of uninfected CD4^+^ T cells following the interaction of the HIV envelope glycoprotein and its co-receptor CXCR4 [24]. On the opposite, HIV infection inhibits autophagy in macrophages, dendritic cells [25], [26], [27] or even in CD4^+^ T cell lines [28].

The damage-regulated autophagy modulator (DRAM), a lysosomal protein, has been reported to link p53 to autophagy [29]. It shows a high degree of conservation throughout evolution. Interestingly, its expression was reported to be lower in certain tumors, suggesting it plays a role in human pathology. However, whether DRAM links p53 and LMP in the context of host-pathogens relationships has never been addressed, to the best of our knowledge.

Our results highlight the major role played by DRAM in the regulation of LMP and autophagy in HIV-infected CD4^+^ T cells downstream from p53 activation. A specific siRNA blocking DRAM protein expression inhibited LMP and prevented the death of CD4^+^ T cells, which leads to a higher number of infected cells. We propose that the ancestral DRAM protein represents a mechanism of self-defense involved in the elimination of microbe-infected cells and constitutes a critical aspect of antiviral immunity.

## Results

### a) HIV-1 infection increases DRAM expression

Based on our previous results [7], [10], we infected purified primary CD4^+^ T cells with HIV-1_LAI_ for 12 h at a MOI of 0.01, then activated the cells with ConA and IL-2. The number of infected cells (intracellularly stained with anti-HIV-Gag antibody) peaked five days after infection (**Figure S1A, B**), and a concomitant increase in lysosomal destabilization, as demonstrated by the release of cathepsin D (B and L, not shown), was observed (**Figure S1C**). Thus, lysosomes are rapidly permeabilized in primary CD4^+^ T lymphocytes infected with HIV-1, resulting in the release of cathepsins into the cytosol [10].

**Figure 1:**
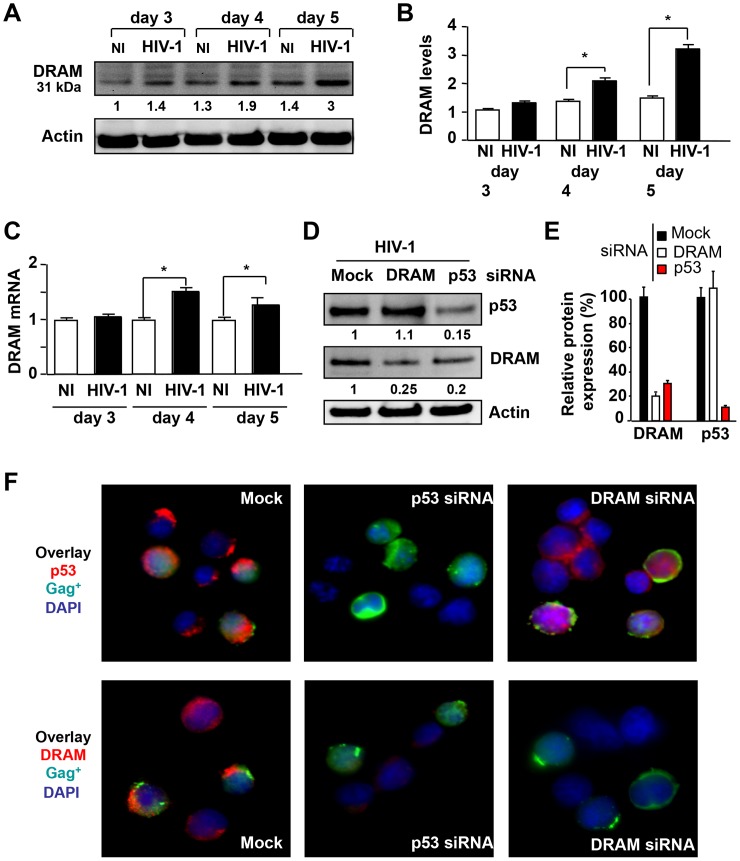
HIV-1 infection induces DRAM expression in CD4^+^ T cells. Primary CD4^+^ T cells in the absence (NI) or presence of HIV-1_LAI_ (HIV-1) have been analyzed. (**A**) Lysates of CD4^+^ T cells were prepared on days 3, 4 and 5 post-infection and separated by western blots, and then immunoblotted with specific antibody against DRAM. Actin was used as a control for protein loading. (**B**) DRAM protein levels were quantified in three independent experiments. Non-infected cells on day 3 were considered arbitrarily equivalent to 1 for normalization. Bars show the mean ± SD. The asterisk (*) indicates statistically significant difference, p<0.05. (**C**) DRAM mRNA level was quantified by RT-PCR on days 3, 4 and 5 post-infection. Non-infected cells were considered arbitrarily equivalent to 1. Bars show the mean ± SD of three independent experiments. The asterisk (*) indicates statistically significant difference, p<0.05. **(D**) HIV-infected CD4^+^ T lymphocytes were transfected with either control siRNA (mock) or siRNAs specific for p53 or DRAM. Cells extracts at day 5 post-transfection were analyzed and immunoblotted with specific p53 and DRAM antibodies. Actin was used as a control of loading. (**E**) Bars show the mean ± SD of four independent experiments. (**F**) Cells were stained with antibodies against p53 or DRAM (in red) or with an antibody against Gag (in green). Nuclei were counterstained with DAPI (blue).

We then assessed the expression of DRAM in primary CD4^+^ T cells infected with HIV-1. We detected more DRAM protein at 31 kDa by western blots at day five in HIV-infected CD4^+^ T cells than in non-infected cells (**Figure 1A, B**). Consistently with previous results [15], [30], we found by western blot that p53 in HIV-1-infected primary CD4^+^ T cells is phosphorylated on serine 15 (P-p53) and increases concomitantly with the increase of viral replication (**Figure S1D**). By cell fractionation, we found an accumulation of p53 within the nucleus (**Figure S1E**). Fluorescent microscopic analysis of P-p53 showed that the percentages of CD4^+^ T cells expressing P-p53 increase (**Figure S1F**) and P- p53 occurred in HIV-infected primary CD4^+^ T cells (Gag^+^) but not in non-infected cells (Gag^−^) (**Figure S1G**). More than 80% of the CD4^+^ T cells expressing P-p53 were positive for Gag antigen. We then assessed mRNA levels of DRAM in HIV-infected primary CD4^+^ T cells versus non-infected cells. Quantitative gene expression analysis with real-time RT-PCR showed that DRAM expression was increased on days 4 and 5 post-infection in HIV-infected CD4^+^ T cells (**Figure 1C**). We also found an up-regulation of the mRNA levels of p53-inducible genes such as Bax, p21, and HDM2 (data not shown). Altogether, these results indicate that HIV infection in primary CD4^+^ T cells causes p53 transcriptional activation associated with increased expression of DRAM.

In order to confirm the link between p53 activation and DRAM expression, we used specific siRNAs targeting DRAM and p53 mRNA. As expected, the inhibition of DRAM protein production had no effect on p53 expression, whereas the siRNA targeting p53 resulted in lower levels of both the p53 and DRAM proteins in HIV-infected primary CD4^+^ T cells (**Figure 1D, E and F**). Interestingly, DRAM (in red) forms aggregates in Gag^+^ cells (in green) (**Figure 1F**). Confocal microscopy revealed the colocalization of DRAM and the lysosome-associated membrane glycoprotein 2 (LAMP2) that confirms its lysosomale localization (**Figure S2**). Quantification of DRAM and LAMP2 expression using ImageJ software indicated that the level of DRAM is increased in HIV-infected CD4^+^ T cells but not of LAMP2. Altogether, our results demonstrated for the first time that HIV-1 induces DRAM expression, which is linked to p53.

### b) DRAM triggers autophagy in HIV-1-infected CD4^+^ T cells

Next, we examined autophagy-related ultrastructures in CD4^+^ T infected by HIV. Electron microscopic analyses of HIV-infected primary CD4^+^ T cells with budding HIV viruses (arrow heads) showed that cells contained large numbers of vacuoles with double-membrane structures (arrows) (**Figure S3A, B**). These vacuoles were not observed in non-infected cells (**Figure S3, panel Aa**). The presence of cytoplasmic material within these double-membrane structures represents autophagosomes (large arrows), the first autophagic-related structures to be produced (**Figure S3, panel Ca, b**). We also observed lysosomes located near autophagosomes (**Figure S3, panel Cb**). Microphotographs also show autophagolysosomes (dashed arrows) resulting from the fusion of lysosomes with autophagosomes associated with degradation of the sequestered content, in HIV-infected CD4^+^ T cells (**Figure S3, panel Cc**). In the latter, autophagolysosomes were more frequently detectable than autophagosome structures (**Figure S3D**). Moreover, in CD4^+^ T cells, in contrary to the situation reported for differentiated macrophages [25] or monocytes infected with HIV at day 5 (**Figure S3E**), we detected no viral particles in autophagosome structures.

**Figure 2:**
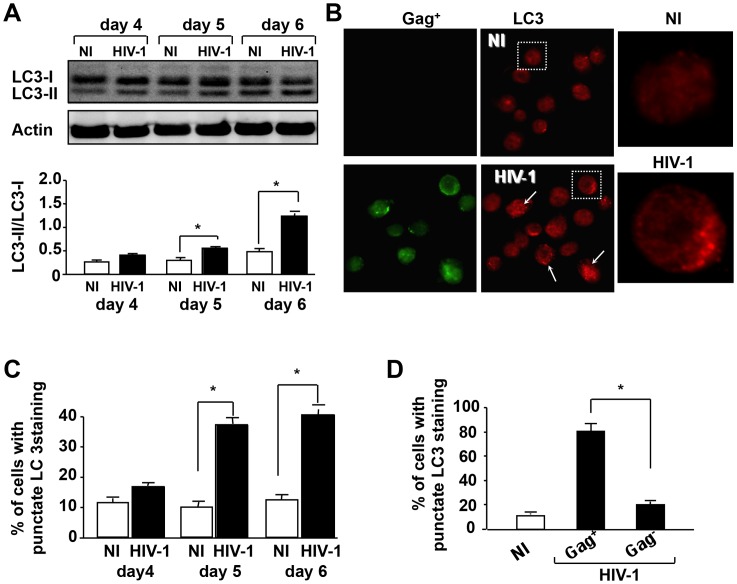
HIV-1 infection induces autophagic machinery in CD4^+^ T cells. (**A**) Western-blot analysis of LC3-I and LC3-II proteins in CD4^+^ T cells in the absence (NI) or presence of HIV-1_LAI_ (HIV-1) on days 4, 5 and 6 post-infection. Actin was used as a control for protein loading. Ratio of LC3-II/LC3-I was calculated and bars show the mean ± SD of four independent experiments; *, p<0.05. (**B**) Cells were stained on day 5 with mAbs recognizing the LC3 antigen (red) and the Gag antigen (green), and examined by confocal microscopy. The insets show magnified images of LC3-II puncta in infected cells. (**C**) Percentages of CD4^+^ T cells displaying punctate LC3-II staining on days 4, 5 and 6 post-infection. Histograms show the means ± SD of five individual experiments; *, p<0.05. More than 200 cells were counted for each condition. (**D**) Within infected samples, quantification of Gag^+^ and Gag^−^ cells with punctate LC3-II accumulation. More than 200 cells were counted for each staining, 5 days post-infection, and the results presented are means ± SD of five independent experiments; *, p<0.05.

The identification of several genes encoding proteins responsible for the execution of autophagy has facilitated the detection and manipulation of the autophagy pathway (see reviews [16], [18]. LC3 is a protein marker reliably associated with autophagosomes during autophagy [31] although its localization in phagosomes after TLR stimulation has also been reported [32]. We investigated LC3 status, to identify the machinery involved in autophagy at the biochemical level. Detection of both LC3-I and LC3-II revealed that the ratio of LC3-II/LC3-I was higher in HIV-infected CD4^+^ T cells than in non-infected CD4^+^ T cells (**Figure 2A**). In order to examine the relationship between Gag^+^ target cells and LC3 proteins, we analyzed the formation of LC3 puncta by confocal microscopy. Consistent with immunoblotting results, larger numbers of CD4^+^ T cells with punctate LC3 were found in HIV-infected cultures (Gag^+^) than in non-infected ones (**Figure 2B, C**). Moreover, we compared LC3 staining in uninfected bystander cells (Gag^−^) and HIV-1 infected CD4^+^ T cells (Gag^+^, green) found within infected samples five days after infection and found that the formation of LC3 puncta was mostly confined to Gag^+^ target cells (**Figure 2D**).

**Figure 3:**
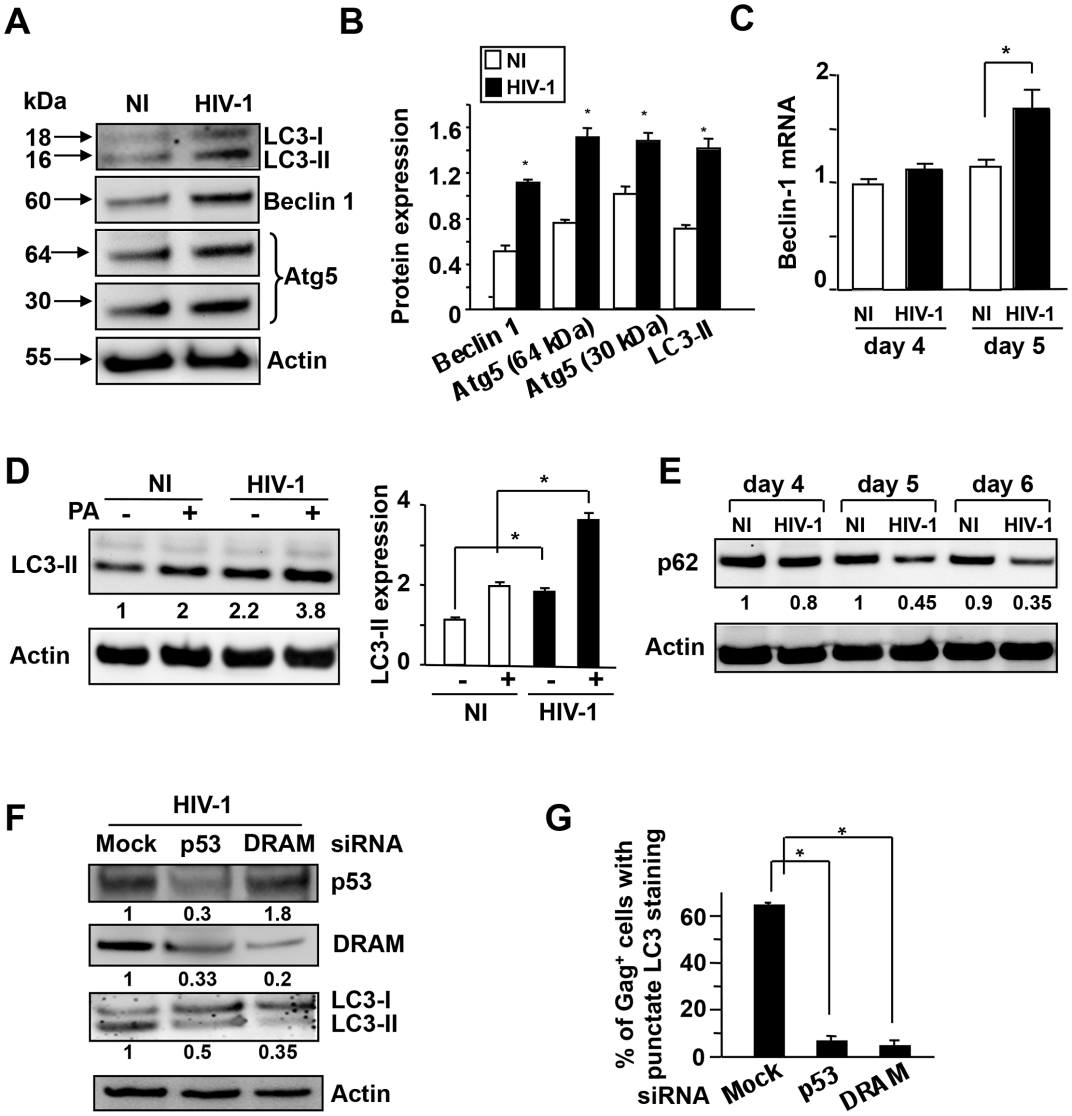
DRAM regulates autophagy in CD4^+^ T cells infected with HIV-1. (**A**) Immunoblots of lysates from CD4^+^ T cells in the absence (NI) or presence of HIV-1_LAI_ (HIV-1) at day 5 after infection. Membranes were probed for LC3, beclin 1, Atg5, and actin. (**B**) The amount of each protein was measured and normalized with respect to the loading control (Actin) (relative expression). Bars show the mean ± SD of five independent experiments. *, p<0.05. (**C**) Beclin 1 mRNA level was quantified by RT-PCR on days 4 and 5 post-infection. Non-infected cells (NI) on day 4 were considered arbitrarily equivalent to 1 for normalization. Bars show the mean ± SD of three independent experiments (*, p<0.05). (**D**) Samples from uninfected (NI) and HIV-infected CD4^+^ T cells incubated in the absence or presence of Pepstatin A (PA). At day 5 the cells were dissolved in SDS. Membranes were probed for LC3-II and actin. Bars show the mean ± SD of three independent experiments; *, p<0.05. (**E**) Western-blot analysis of p62 protein in CD4^+^ T cells in the absence (NI) or presence of HIV-1_LAI_ (HIV-1) on days 4, 5 and 6 post-infection. Actin was used as a control for protein loading. The level of p62 was calculated, and the levels in non-infected cells (NI) on day 4 were considered arbitrarily equivalent to 1. (**F**) CD4^+^ T cells were transfected with siRNA specific for p53 and DRAM or the control siRNA (mock) and then infected with HIV-1. On day 5 post-infection, cells were analyzed by immunoblotting using LC-3 antibody. Actin was a control of loading. Values represent LC3-II/LC3-I ratio. (**G**) LC3-II staining (number of puncta per cell ≥6) was examined by fluorescence microscopy in Gag^+^ target cells. The values shown are means ± SD of three independent experiments (≥200 cells were examined per experiment).

We next analyzed the early events associated with the autophagy pathway. The initiation of autophagy involves a complex of Beclin 1 and PIK3C3, whereas Atg5 (30 kDa) is required for autophagosome precursor synthesis. Atg5 forms a complex with Atg12 (the Atg5-Atg12 complex), which has a molecular weight of 64 kDa and participates in the autophagosome membrane elongation. Consistent with the accumulation of LC3-II in HIV-infected CD4^+^ T cells, on day 5 post-infection, Beclin 1 protein level was higher in HIV-infected CD4^+^ T cells than in uninfected CD4^+^ T cells (**Figure 3A, B**). We also found that the expression of Beclin mRNA is significantly increased on day 5 post-infection (**Figure 3C**), whereas the expression of DRAM mRNA increases at day 4 (**Figure 1C**). Moreover, the Atg5 protein level was higher in HIV-infected CD4^+^ T cells than in non-infected ones, and a band at 64 kDa band corresponding to the Atg5/Atg12 complex was detected (**Figure 3A, B**). Using small interfering RNAs (siRNAs) directed against the mRNAs for BECLIN1 and ATG5, LC3-II protein levels were lower, as expected, in HIV-1-infected CD4^+^ T cells than in control siRNA (mock)-transfected cells, as shown by western blotting and fluorescence microscopy (**Figure S4A, B**). Altogether, these results support the hypothesis that HIV infection of primary CD4^+^ T cells induces autophagy.

It has been reported that depletion of LAMP2 by siRNA leads to the accumulation of autophagic vacuoles [33], [34]. Thus, the accumulation of autophagic vacuoles in HIV-infected CD4 T cells could be the consequence of LAMP depletion. However, confocal microscopy of LAMP2 in HIV-infected cells revealed no major difference with uninfected CD4^+^ T cells (**Figure S2**). Now, the appearance of autophagosomes can result from an increase in their induction or, since virtually all cells have a basal autophagic rate, from a block of their turnover. We used Pepstatin A (PA) to inhibit lysosomal proteases in order to influence the lysosome-autophagy pathway and block the flux of autophagy. We detected increased protein levels of LC3-II in HIV-infected primary CD4^+^ T cells treated with PA (**Figure 3D**). One of the best-characterized substrates of selective autophagy is p62 (sequestosome 1/SQSTM1). The p62 protein binds to the autophagy regulator Atg8/LC3 and is incorporated into the autophagosome. Lysosomal degradation of autophagosomes leads to a decrease in p62 levels during autophagy [35], [36]; conversely, impairment of autophagy is accompanied by the accumulation of p62 [37]. Thus, we measured the expression of p62 as a marker of the terminal step in the autophagy pathway. We found that the amount of p62 decreased in HIV-infected CD4^+^ T cells (**Figure 3E**). These results show that autophagy results more from induction of autophagosomes rather than from reduced autophagy flux in HIV-infected CD4^+^ T cells.

Finally, we investigated whether knocking down DRAM and p53 expressions inhibits autophagy in HIV-infected CD4^+^ T cells. Immunoblotting showed that the inhibition of DRAM and p53 protein production decreased the formation of LC3-II (**Figure 3F**). Moreover, staining for LC3 remained punctuate in Gag^+^ CD4^+^ T cells treated with control siRNA (mock), whereas lower levels of puncta were displayed after treatment with siRNA targeting DRAM or p53 (**Figure 3G**). Altogether these results demonstrated that HIV infection of primary CD4^+^ T cells mediates autophagy in a DRAM/p53 dependent pathway.

### c) DRAM triggers lysosomal destabilization in HIV-1-infected CD4^+^ T cells

**Figure 4:**
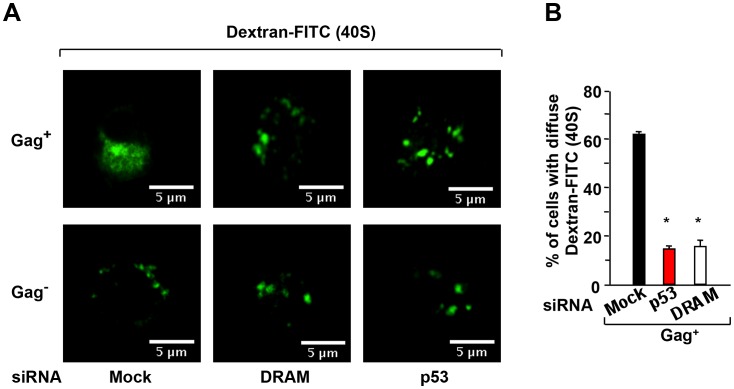
DRAM induces lysosomal membrane permeabilization (LMP) in HIV-infected CD4 T cells. CD4^+^ T cells were transfected with siRNA specific for p53 and DRAM or the control siRNA (mock). (**A**) Cells were loaded with fluorescent dextran molecules of 40 kDa and examined by laser scanning confocal microscopy. Pictures show gag^+^ and gag^−^ cells from infected samples. Diffuse staining revealed lysosomal permeabilization. (**B**) The values shown are means ± SD of two independent experiments (≥200 cells were examined).

**Figure 5:**
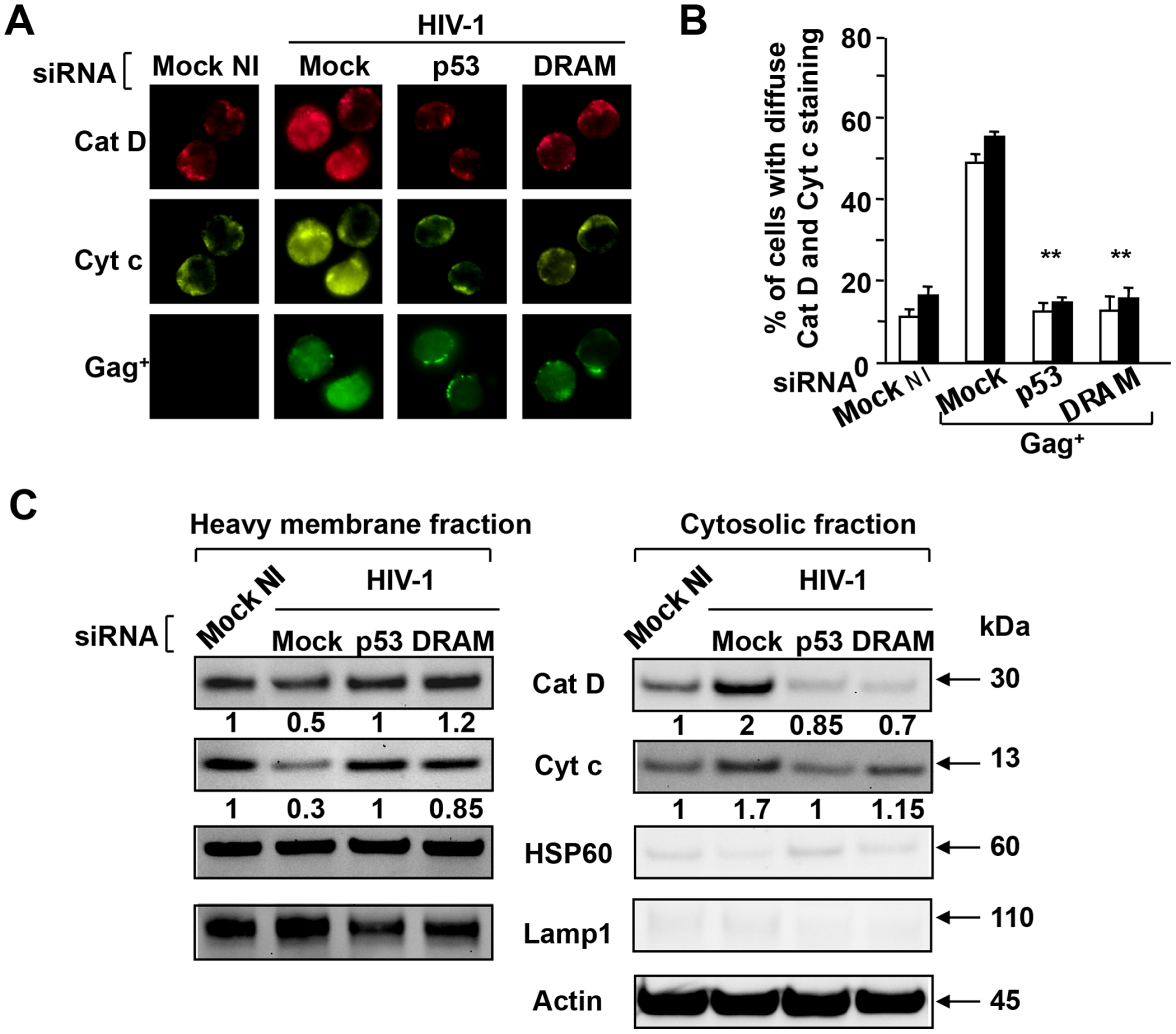
DRAM induces cathepsin D and cytochrome C release in HIV-infected CD4^+^ T cells. HIV-infected CD4^+^ T cells were transfected with siRNA specific for p53 and DRAM or the control siRNA (mock) and then infected with HIV-1. Non-infected cells were used as a control. (**A**) At day 5 post-infection, cells were stained with specific antibodies against cathepsin D (Cat D), cytochrome C (Cyt c) and Gag antigen. (**B**) The subcellular distribution of Cat D and Cyt c in the Gag^+^ cells was analyzed. More than 200 cells were counted for each staining and the results shown are the means ± SD of three independent experiments; * p<0.05. (**C**) Heavy membrane and cytosolic fractions derived from CD4^+^ T cells, five days after infection, were analyzed by western blotting for the presence of Cat D and Cyt c. Anti-HSP60 and anti-Lamp-1 were used to verify the absence of mitochondrial and lysosomal contamination, respectively. Actin was used as a control of loading. One representative experiment out to three performed giving similar results is shown.

Because we have previously showed that HIV mediates LMP, we investigated whether the knockdown of DRAM and p53 affected LMP. First, to monitor lysosomal destabilisation, CD4^+^ T cells isolated from HIV-1-infected cultures at day 5 were loaded with FITC-conjugated dextran of 40-kDa, and after a 2-h chase, the cells were visualized by laser scanning confocal microscopy (**Figure 4A, B**). The redistribution of the FITC-dextran molecules was observed in Gag^+^ cells (diffuse staining), but not in Gag^−^ cells (**Figure 4A**). Thus, a diffuse staining pattern for the 40-kDa FITC-dextran molecules was observed in 60% of the Gag^+^ cells (≥200 cells were examined) (**Figure 4B**), which is indicative of lysosomal efflux. By inhibiting the expression of DRAM and p53 using siRNAs, the 40-kDa molecules were almost totally confined to punctate structures, consistent with exclusive lysosomal localization (**Figure 4A, B**). We then assessed by confocal microscopy whether the inhibition of p53 and DRAM prevented cathepsin D release in the cytosol. By modifying the levels of p53 and DRAM proteins with specific siRNAs, we showed a significant decrease of cathepsin D release in cytosol in Gag^+^ target cells (**Figure 5A, B**). Digitonin-based subcellular fractionation and immunoblot analysis was used to visualize cathepsin D release. Our results showed higher amount of cathepsin D in the cytosol of HIV-1 CD4 T cells in comparison to uninfected cells. In presence of specific siRNAs against p53 and DRAM, cathepsin D release is reduced (**Figure 5C**). Anti-Lamp-1 was used to verify the absence of lysosomal contamination in the cytosolic fraction.

**Figure 6:**
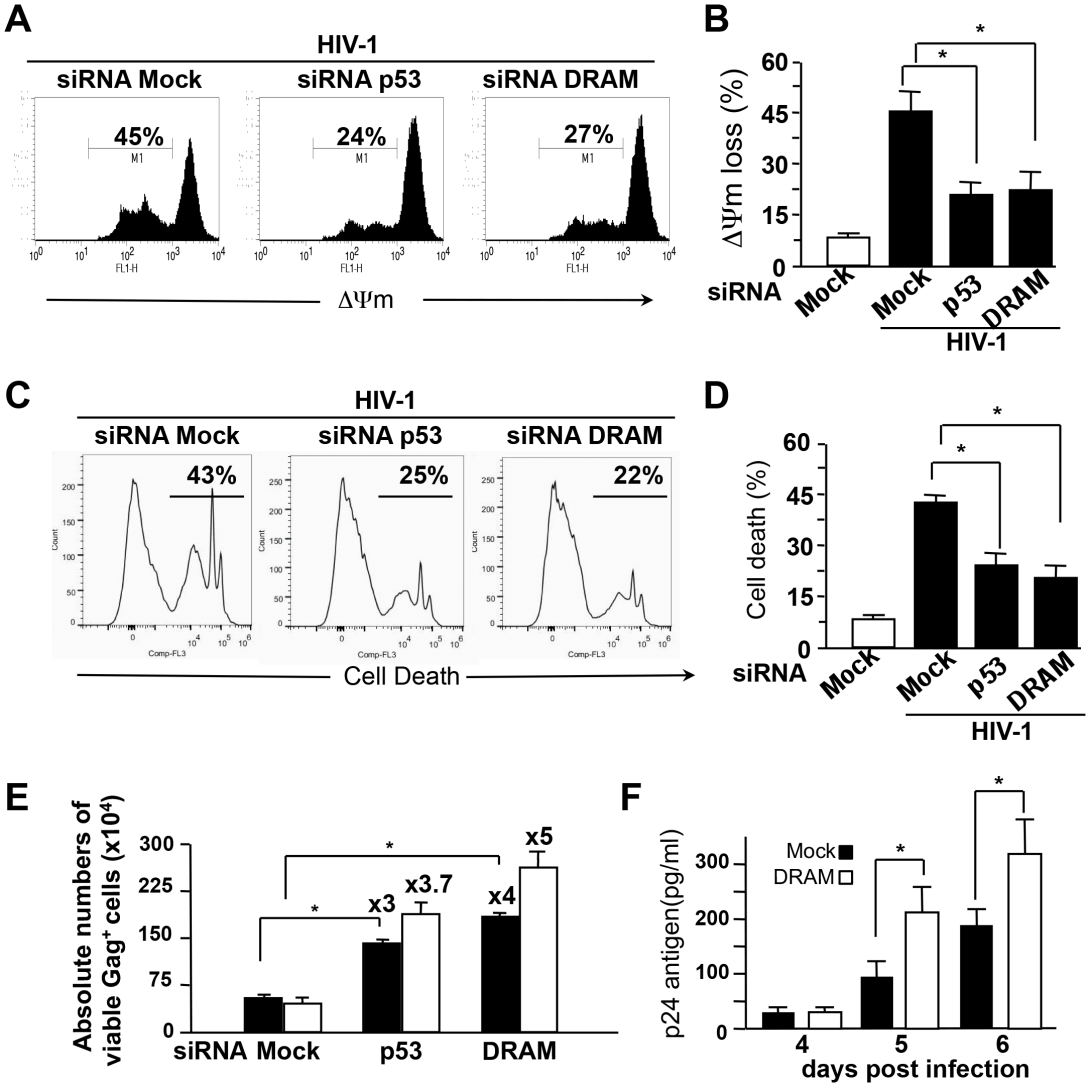
DRAM induces cell death in HIV-infected CD4^+^ T cells. CD4^+^ T cells were transfected with siRNAs specific for p53 or DRAM or the control siRNA (mock), and then infected with HIV. (**A**) ΔΨm loss was assessed using a DioC_6_ probe at day 5 post-infection. A representative experiment out five independent experiments is shown. (**B**) The values shown are means ± SD; *, p<0.05. (**C**) Cell death was assessed using a propidium iodide (PI) probe at day 5 post-infection. A representative experiment is shown. (**D**) The values shown are means ± SD of three independent experiments; *, p<0.05. (**E**) Absolute numbers of infected cells were calculated on days 5 (black square) and 6 (white square) post-infection. Histograms show means ± SD of three independent experiments; * p<0.05. (**F**) Viral production was followed by measuring p24 release in the supernatants of CD4^+^ T cells. Results are the mean ± SD of three independent experiments; * p<0.05.

We previously reported that cathepsin D release from lysosomes is an early event resulting in mitochondrial destabilization and cytochrome C release in HIV-1-infected CD4^+^ T cells [10]. This led us to investigate whether the knockdown of DRAM and p53 affected MOMP. Our results clearly show that siRNAs specific for DRAM and p53 inhibited cytochrome C release in Gag^+^ as assessed by fluorescence microscopy (**Figure 5A, B**) and by immunoblot (**Figure 5C**). Moreover, MOMP (ΔΨm) and cell death (PI^+^) assessed by flow cytometry using DioC_6_ probe (**Figure 6A, B** **)** and using propidium iodide (**Figure 6C, D** **)**, respectively, confirms a preventive effect of specific siRNA for DRAM and p53. We also evaluated whether the inhibition the Beclin 1 and Atg5 gene products impacts on LMP. In fact, as demonstrated by the diffuse staining of cathepsin D in Gag^+^ cells, inhibition of BECLIN1 and ATG5 expression by specific siRNAs had no major effect on LMP (**Figure S5A, B**) and cell death (**Figure S5C**). Because it has been proposed that HIV mediates death of bystander CD4 T cells through autophagy [24], we incubated quiescent CD4 T cells in the presence of HIV and ddI. At days 4 and 5, we analyzed mitochondrial depolarization (ΔΨm), cell death (PI^+^) and viral replication (Gag detection) by flow cytometry (**Figure S6**). In the absence of viral replication (less than 2% of the CD4^+^ T cells are Gag^+^) the cells do not undergo death [7], [10]. We also demonstrated by western blot the absence of DRAM augmentation and the absence of LC3-II in bystander cells in contrast to what happen in productively infected CD4^+^ T cells (**Figure S6**).

HIV-1 accessory proteins such as the viral protein R (Vpr) or nef have been associated with T cell death. Thus, Vpr causes G2 arrest and apoptosis in cycling cells [38], [39]. Therefore, we assessed whether Vpr modulated LMP. We infected purified CD4^+^ T cells with either wild type or a Vpr-defective NL4-3 virus. Our results showed that the release of cathepsin D and cytochrome C is similar in the presence or absence of Vpr, and that inhibition of DRAM using specific siRNA prevents LMP (cathepsin D release, **Figure S7B, C**) and MOMP (cytochrome c release, **Figure S7B**). Thus, Vpr encoded by HIV is dispensable to trigger LMP in primary CD4^+^ T cells. Another viral protein, Nef, induces in CD4^+^ T lymphocytes the accumulation of lysosomes [40] and a limited lysosomal permeabilization [10]. Therefore, we assessed whether the inhibition of DRAM and p53 prevents Nef-mediated LMP and MOMP [10]. Whereas overexpression of Nef induced cathepsin D and cytochrome C releases, in contrast to Nef antisense (**Figure S8A, B**), siRNA against DRAM or p53 did not impact LMP (**Figure S8A, B**) and MOMP (**Figure S8C**) mediated by Nef. However, we cannot exclude that residual DRAM is enough to induce LMP.

Because LMP is upstream from MOMP and cell death, we evaluated the consequence of DRAM inhibition on viral infection. Thus, we evaluated the numbers of infected cells (Gag^+^) in the culture as previously described [10]. The siRNAs targeting p53 and DRAM clearly resulted in the presence of greater number of HIV-infected CD4^+^ T cells than control siRNA (mock) (**Figure 6E**). This is consistent with the levels of HIV DNA measured (data not shown). Thus, the knock-down of DRAM and p53 protein levels by specific siRNAs resulted in numbers of infected cells three to four times greater than the control. We also measured by ELISA in the supernatants the p24 production in the presence or absence of DRAM. Our results revealed that viral production is increased following DRAM knock-down as compared to the control (**Figure 6F** **)**.

Taken together our results demonstrate the major role for DRAM in regulating LMP and cell death in HIV-infected cells.

## Discussion

Many different viruses induce lysosomal damage and kill the cells in which they replicate [41], [42], [43], [44]. Our results provide new insight in virus-cell interaction, showing a clear relationship between p53 activation, DRAM and LMP in a model of viral replication-mediated cell death. The data presented here demonstrate for the first time that DRAM controls LMP; the depletion of p53 and DRAM indeed prevents LMP and cell death in HIV-infected CD4^+^ T cells. Thus, this provides a rationale for the previous observation that the tumor suppressor p53 can trigger a lysosomal destabilization that contributes to cell death [11], [12].

These results reinforce the concept that productive HIV infection is associated with a caspase-independent cell death pathway associated with early lysosomal destabilization. The discovery of the role of DRAM during HIV infection identifies this molecule as a new regulator of host cell-pathogen interactions, contributing to the control of viral infection. This may represent a process of altruistic cell suicide developed by multicellular organisms to defend themselves against microbial infections.

Lysosomes are permeabilized in CD4^+^ T lymphocytes productively infected with HIV-1, resulting in the early release of cathepsins into the cytosol. The released cathepsin D acts upstream from the conformational change in Bax and MOMP [10]. On the contrary, bystander cells exposed to HIV do not express higher levels of DRAM and LC3, in agreement with idea that viral replication induces a death signal [5], [6], [7], [10]. Accumulating evidence indicates that lysosomes function as death signal integrators in response to a wide variety of death stimuli [1]. We demonstrated that the inhibition of DRAM by specific siRNA prevents cathepsin D release, demonstrating for the first time that DRAM is critical for LMP in the context of host-pathogen interaction. We found higher levels of both DRAM mRNA and protein in HIV-infected CD4^+^ T cells than in non-infected cells. However, it was previously shown that the overexpression of DRAM was not enough for inducing autophagy and cell death [29]. This initial observation suggests that additional partners and/or translational modifications are probably required for DRAM-mediated LMP in HIV-infected CD4^+^ T cells. We found higher amounts of DRAM that form puncta in Gag^+^ cells, as visualized by fluorescence microscopy. Bax and Bak, the gatekeepers that induce MOMP, also form aggregates. Unfortunately, attempts at immunoprecipitating DRAM in primary CD4^+^ T cells infected by HIV using commercial antibodies to identify partners have been unsuccessful so far. Therefore, further analysis is necessary to specify the mechanism by which DRAM destabilizes lysosome membranes in primary CD4^+^ T cells.

Our results demonstrated that inhibition of p53 by specific siRNA prevent DRAM expression and autophagy. The infection of T-cell lines and primary CD4^+^ T cells with HIV was initially reported to be associated with stronger expression of pro-apoptotic genes, such as those encoding Bax, p21 and MDM2 [45], [46]. CD4^+^ T cells productively infected with HIV display phosphorylation of p53 on serine 15 leading to the accumulation of p53 within the nucleus in agreement with other observations [15], [30]. However, in these models the role of p53 was not directly addressed. Here, we demonstrated using siRNAs that inhibition of p53 prevents LMP and programmed cell death in productively infected CD4^+^ T cells. Viral accessory protein Vpr has been proposed to induce p53 phosphorylation [38], and it causes G2 arrest and apoptosis via ATR [39]. However, resting CD4^+^ T cells infected by HIV and stimulated with mitogens accumulate predominantly in the G_0_/G1 phase of the cell cycle at day 5 [7], [10], [47]. Consistently with the observation in cell cycle in primary CD4^+^ T cells, our data indicate that Vpr is dispensable for LMP. Other candidates encoded by HIV such as the gp120 envelope glycoprotein [48] have been proposed to induce p53 phosphorylation. However, these proteins in primary CD4^+^ T cells are dispensable for HIV infection-mediated cell death [9], [47] and no syncytia are observed in the culture [7], [10], [47]. Moreover, in bystander CD4^+^ T cells, DRAM and LC3-II are not increased.

Nef is in itself an important pathogenic factor in HIV-1 infection. In severe combined immunodeficient (SCID)–hu (thymus/liver) mice, Nef is pathogenic, as evidenced by thymocyte depletion [49]. It has also been reported that Nef can induce in CD4^+^ T lymphocytes the accumulation of lysosomes [40] and a limited lysosomal permeabilization [10]. Here, we found that DRAM or p53 siRNA has no preventive effect on LMP-mediated by Nef. In agreement with the absence of a p53 effect, overexpression of Nef is not associated with p53 activation (data not shown). Previous observations have indicated that Nef, instead of activating p53, inhibits it [50], [51]. Nef is a critical factor that enhances virus replication *in vitro* in primary CD4^+^ T cells and is clearly associated with AIDS [52], [53], [54], [55], [56]. Thus, it is conceivable that Nef indirectly acts as a cofactor in the onset of the permeabilization of lysosomes by favoring viral replication [10]. Activation of p53 – the guardian of the genome – has been reported as a sensor detecting pathogen replication in order to eliminate infected cells [14]. Therefore, prompt induction of LMP and apoptosis of virally-infected cells via p53/DRAM activation will be beneficial for the host as an altruistic cell suicide to limit virus dissemination.

We demonstrated that DRAM regulated autophagy in virally-infected CD4^+^ T cells downstream from active p53. Thus, whereas DRAM was initially described as regulating autophagy in cancer [29], we extended here its role in the context of host-pathogen interactions. Within HIV-infected CD4^+^ T cells, we have observed (i) ultrastructural autophagy-associated structures, such as autophagosomes and autophagolysosomes; (ii) higher levels of Beclin 1, which is essential for the induction of autophagy; (iii) Atg5-Atg12 complexes, which are considered essential for membrane elongation, (iv) LC3-II aggregates in p24^+^ cells, involved in the formation of the autophagosome membrane, and (v) the degradation of p62. These results suggest that HIV mediates autophagy flux in primary CD4^+^ T cells. It has been suggested that HIV infection inhibits starvation-induced or rapamycin-induced autophagy in CD4 T cell lines [28]. Thus, using primary CD4 T cells and in the absence of additional stimuli to trigger autophagy, we demonstrated that HIV infection increased autophagy instead of inhibiting it. In macrophages infected with HIV, the virus inhibits autophagy flux through Nef, leading to the accumulation of autophagic vacuoles [25]. Thus, although autophagy is observed in both cellular models, the mechanisms are distinct. Indeed, macrophages are resistant to apoptosis, contrary to infected CD4^+^ T cells. Autophagy is suspected of participating in the elimination of damaged organelles and preserving cell integrity from the accumulation of abnormal proteins [21]. Thus, autophagy could be induced to limit damage induced by disrupted lysosomes. The clearance of ruptured lysosomes by autophagy has been previously shown [57]. Our results demonstrate in primary CD4^+^ T cells that siRNAs blocking the expression of the Atg5 and Beclin 1 proteins have no effect on LMP and cathepsin D release. Therefore, the induction of autophagy is not directly responsible for the lethality of HIV-infected CD4^+^ T cells. Viral protein clearance requires intact lysosomes to remove protein aggregates and limit cell death [58]. Herein, by preserving LMP by knocking down DRAM, we prevented cell death mediated by HIV infection. Interestingly, we found greater viral infection and production. Altogether, these data demonstrate that although DRAM is controlling both autophagy and LMP, only this latter process is essential for survival of the infected cells. Viral replication is thought to directly induce CD4 T-cell death during the acute phase of HIV infection, particularly in the intestine [59], [60], [61], [62]. This suggests that an absence of DRAM could be favorable for viral dissemination and persistence of virally-infected CD4^+^ T-cells in HIV-infected individuals. Pharmacological intervention to modulate DRAM in HIV-infected CD4^+^ T cells may thus help to eliminate viral reservoirs and delay development of clinical AIDS.

Our results demonstrate for the first time that the destabilization of lysosomes is induced by DRAM and is an early event in the commitment to cell death contributing in the control of viral infection.

## Methods

### Isolation of lymphocytes and viral infection

PBMC were isolated from the peripheral blood of anonymous healthy volunteers. All blood donors were informed and agreed to a written consent prior to blood donation in accordance with the guidelines of the Etablissement Français du Sang. CD4^+^ T cells were obtained by negative selection with a CD4 T-cell Isolation kit (Miltenyi Biotec). The CD4^+^ T-cell preparation was at least 98% pure. Monocytes (5%) were added to the purified cells, to ensure full T-cell activation. The cells were incubated with HIV-1_LAI_ for 12 h at a multiplicity of infection (MOI) of 0.01, and activated with 1 µg/ml ConA (Sigma-Aldrich) and 100 units/ml recombinant human IL-2 (Roussel-Uclaf, France), as previously described [7], [10]. The virus was produced from CEM-LTRgfp that allows to monitoring viral replication [63]. HIV-1_LAI_ was recovered from supernatant at the peak of replication. Virus stocks were prepared by transfection of 293T cells with plasmids of Wt and Vpr-defective NL4-3 (kindly provided by S. Benichou). HIV replication was assessed by monitoring the Gag^+^ cells by flow cytometry using anti-HIV-Gag (KC57-PE, Beckman-Coulter) and by measuring Gag-p24 release in supernatants by an Innotest HIV antigen enzyme-linked immunosorbent assay (ELISA) kit (Ingen). The number of live infected CD4^+^ T cells was assessed by flow cytometry as previously described [7], [10], [64], [65]. Dead cells were detected using 1 µM of propidium iodide (PI) from Molecular Probes and Annexin V-FITC from Beckman Coulter. To evaluate changes in the inner mitochondrial transmembrane potential ΔΨm, cells were stained for 15 min at 37°C with 40 nM of the potential-sensitive fluorescent dye DiOC_6_ (3.3′-diethyloxacarbocyanine) from Molecular Probes.

### Immunofluorescence analyses

The reagents used for immunofluorescence studies were: rabbit polyclonal antibodies recognizing anti-MAP LC3 (H-50) purchased from Santa Cruz, anti phospho-p53 (Ser 15) antibodies purchased from Cell Signaling Technology, anti-DRAM antibodies purchased from φProSci, anti-cathepsin D antibodies from Zymed Laboratories, anti-p53 mAb (DO-1) purchased from Santa Cruz, anti-Lamp-2 mAb from Calbiochem, and a sheep anti-cytochrome c antiserum from Sigma. Intracellular Gag antigen was assessed by flow cytometry after fixation and permeabilization of the cells (Intraprep permeabilization reagent, Coulter), which were then stained with FITC- or RD1-labeled mAb against p24^gag^ antigen (KC-57, Beckman coulter). Otherwise, the cells were fixed by incubation with 1% paraformaldehyde, span on glass slides, washed with PBS, and permeabilized by incubation with 0.05% Triton X-100. The cells were washed and incubated with the antibodies indicated in PBS supplemented with 0.5% BSA and 2% FCS. The cells were stained with an Alexa-conjugated secondary antibody (Molecular Probes). Nuclei were counterstained for 5 minutes with 5 µM DAPI (Molecular Probes). The cells were examined by conventional or confocal fluorescence microscopy (Zeiss Microsystems). For the formation of LC3-II aggregates, cells with more than 6±2 vesicles were considered positive as previously described [31]. To monitor lysosomal destabilization, CD4^+^ T cells were incubated for 2 h with 5 mg/ml FITC-dextran of 40-kDa (Sigma) as previously described [10]. After a 2-h chase period, the cells were stained with anti-p24 mAb (Gag^+^) and examined by laser scanning confocal microscopy.

### Immunoblotting

Pellets of 1×10^6^ CD4^+^ T cells were either directly resuspended in Laemmli buffer containing 2% SDS and 10% 2-ME and boiled for 5 minutes, or lysed in Nonidet P-40 buffer (1% NP-40, 50 mM Tris-HCl (pH 7.4), 150 mM NaCl) supplemented with protease inhibitors. Pepstatin was purchased from Sigma. Cytosolic and nuclear fractions were obtained by extraction with the NE-PER kit (Nuclear and Cytoplasmic Extraction Reagents from PIERCE). Lysates were then subjected to electrophoresis in NUPAGE 4–20% polyacrylamide gels (Invitrogene). The proteins were transferred to polyvinylidene difluoride membranes (Amersham Bioscience) and then incubated with primary antibodies and with horseradish peroxidase-coupled secondary reagents (Amersham Biosciences). The primary antibodies used for western blotting were: rabbit antisera against Beclin 1 (H-300, Santa Cruz), phospho-p53 (Ser 15) (Cell Signaling Technology), DRAM (Stressgen), Atg5 (Novus) and tubulin (Santa-Cruz); mouse mAbs against p53 (DO-1, Santa Cruz), Cathepsin D (BD Transduction Laboratories), Cytochrome c (Pharmingen), p62 (Cell Signaling Technology), Lamp-1 (BD Transduction Laboratories), lamin B (Ab-1, Oncogene Research Products Calbiochem) and actin (Millipore). Rabbit antisera against MAP LC3 was purchased from MBL. Productive HIV infection was visualized by western blotting that allows detection of the presence of viral antigens in cell extracts. The immunoblots were incubated with sera obtained from a pool of HIV-infected patients, and then revealed with horseradish peroxidase-linked goat anti-human secondary antibodies (Amersham Biosciences). The blots were then developed by enhanced chemiluminescence methods (ECL^+^ from GE Healthcare), photographed with a CCD camera (GBOX, SYNGENE), and the optical densities measured and normalized with respect to the loading control (tubulin or Hsp60 for mitochondrial fractions). It must be noted that we have tested all commercial antibodies directed against DRAM for the immunoprecipitation assay; none of them are functional.

### Subcellular fractionation

Cytosolic and heavy membrane fractions were generated from 10^7^ cells, using a selective digitonin-based permeabilization and subcellular-fractionation technique as previously described [7], [10]. In brief, CD4 T lymphocytes were washed with cold PBS and then suspended in a lysis buffer (250 mM sucrose, 20 mM Hepes, 5 mM MgCl2, 10 mM KCl, 1 mM EDTA, 1 mM EGTA, pH 7.4, supplemented with protease inhibitors purchased from Roche). The digitonin concentration was 35 µg/ml. After 5 min, the cells were centrifuged and the supernatant was removed (cytosolic fraction). The remaining pellet was resuspended in lysis buffer (150 mM NaCl, 1.0% NP-40, 0.5% deoxycholate, 0.1% SDS, 50 mM Tris-HCl, pH 8.0) and incubated 30 min. The supernatant comprising the membrane fraction was retained after centrifugation (30 min at 15000 g).

### Transfection experiments

Predesigned small interfering RNA (siRNA) molecules targeting p53, DRAM, BECLIN 1 and ATG5 were synthesized by Dharmacon. Scrambled controls were also used. Gene expression was silenced by the small interfering RNA (siRNA) technique [10], using duplexes of 21-nucleotide siRNAs with two 3′-overhanging TT residues (Proligo). The sense strand of the siRNA used to silence the BECLIN 1 gene was CAGTTTGGCACAATCAATATT, that of ATG5 gene seq 1 was GCAACTCTGGATGGGATTGTT and that of seq 2 was CATCTGAGCTACCCGGATATT, whereas that of the DRAM gene was CCACAGAAATCAATGGTGATT. For p53 we used a smart pool. Purified resting CD4^+^ T cells were transfected, by electroporation with siRNAs (0.75 µM/4×10^6^ cells), mediated by the Nucleofection system (Amaxa). Cells were allowed to rest for 16 hours, exposed to HIV-1 cultured for an additional 12 h and then stimulated with Con A and IL-2. We also used the autophagy inhibitor 3-methyladenine in some experiments, but the results obtained were unconclusive, due to a strong toxicity of this drug in primary cultures of human cells.

### Transfection of CD4^+^ T lymphocytes with Nef expression vectors

Plasmids carrying the Nef gene (from the LAI isolate) in a sense (Nef-WT) and antisense (Nef-AS), under the control of the cytomegalovirus promoter, were kindly provided by O. Schwartz (55). CD4^+^ T cells were first transfected with siRNA directed against DRAM and p53, and after overnight culture were restimulated with ConA and IL-2 for 4 days. Thereafter, the cells were transfected with either Nef-WT or Nef-AS using the Nucleofection system (Amaxa). After 24 h, the cells were washed and fixed for flow cytometry and confocal microscopy.

### Real-time RT-PCR analysis

RNAs were isolated from uninfected and HIV-1 infected CD4^+^ T-cells at days 3, 4 and 5 post-infection. cDNA synthesis was performed using the AffinityScript QPCR cDNA Synthesis Kit (Stratagene, Agilent Technologies) with 1 µg of total RNA and random primers. The resulting RT product was expanded using the Brilliant II SYBR Green QPCR Master Mix (Stratagene, Agilent Technologies) and specific primers for DRAM (forward: 5′-AGACTCCATCTTTTCACCCAAA-3′, reverse: 5′-GCTCTTCACCTTTCAAGCCTAA-3′), Beclin-1 (forward: 5′-AAGACAGAGCGATGGTAG-3′, reverse: 5′-CTGGGCTGTGGTAAGTAA-3′) and the housekeeping gene ribosomal RNA S14 subunit (forward: 5′-GGCAGACCGAGATGAATCCTCA-3′, reverse: 5′-CAGGTCCAGGGGTCTTGGTCC-3′). Detection was performed with the Mx3000P QPCR System (Stratagene, Agilent Technologies). Threshold cycle (Ct) values were obtained for each gene at the different time points using the instrument software. Differences in the levels of gene expression over time were determined for each condition by relative quantification using the Delta Ct method.

### Electron microscopy

Pellets of uninfected or infected CD4^+^ T cells were fixed by incubation for 1 h in phosphate buffer pH 7.2 supplemented with 1.6% glutaraldehyde and were then post-fixed by incubation for 2 h in 0.1 M phosphate buffer supplemented with 1% osmium tetroxide. Pieces of cell pellet were washed for five minutes in water and then dehydrated in a series of increasing concentrations of ethanol before embedding in Epon 812 [66]. Ultrathin sections were cut and stained with 4% uranyl acetate and lead citrate. They were then examined under a ZEISS 902 electron microscope, at 80 KV, or under a FEI Technaï 12 microscope at 80 KV.

### Statistical analysis

Data are reported as means ± SEM. The significance of differences was assessed by Student's t test (Prism software) with p<0.05 considered significant.

## Supporting Information

Figure S1 **HIV-1 infection induces p53 activation in CD4^+^ T cells.** CD4^+^ T cells in the absence (NI) or presence of HIV-1_LAI_ (HIV-1) have been analyzed. (**A**) Flow cytometric analyses of HIV-1 Gag antigen expression (Gag-PE) at day 5 after infection. (**B**) Percentage of Gag^+^ cells on days 3, 4 and 5 after infection. Histograms are means ± SD of 10 individual experiments. (**C**) Percentages of cells releasing cathepsin D (Cat D) in CD4^+^ T-cells. Cells (Gag^+^ and Gag^-^) were analyzed by confocal microscopy, after staining for cathepsin D. Results expressed as the mean ± SD of 10 individual experiments. In each condition 100 cells were counted. (**D**) Nuclear fractions from CD4^+^ T cells were analyzed for P-p53 on days 3, 4 and 5 post-infection. Antibody against lamin B was used as a control for protein loading. A typical experiment out of four is shown on the left, and the means ± SD are shown in histograms on the right. *, p<0.05. (**E**) Nuclear and cytoplasmic fractions from CD4^+^ T cells were analyzed for p53 on day 5 post-infection. (**F**) Cells on day 5 were stained with mAbs against P-p53 (red) and p24 antigen (green) and analyzed by fluorescent microscopy. Nuclei were counterstained with DAPI (blue). Representative cells are shown and, in (**G**), a histogram shows the percentages of cells that are P-p53^+^ on days 3, 4, and 5. Results expressed as the mean ± SD of 4 individual experiments. In each condition 100 cells were analyzed. *, p<0.05.
(TIF)

Figure S2 **Colocalization of Lamp-2 and DRAM in infected CD4^+^ T cells.** CD4^+^ T cells are infected with HIV-1 and stained on day 5 post-infection for LAMP2 (green) and DRAM (red). (A) Gag^+^ and Gag^−^ (NI) cells are shown. (B, C) Quantification of DRAM and LAMP2 expressions was assessed using ImageJ software. For each cell, area and pixel value statistics were calculated accordingly and mean fluorescence intensity per cell is shown. Results expressed as the mean ± SD of 2 individual experiments. In each condition 100 cells were analyzed. *, p<0.05.
(TIF)

Figure S3 **Autophagy-related ultrastructures in CD4^+^ T infected by HIV.** (**A**) **a, b** Electron microscopy analyses of autophagy-related ultrastructures in CD4^+^ T cells in the absence (NI) or presence of HIV-1_LAI_ (HIV-1); (**c**) higher magnification of the inset in (**b**); arrows indicate autophagosomes with double-membrane-structures in cells with HIV-1 particles budding at the surface. (**B**) Quantitation of CD4^+^ T cells displaying autophagic vacuoles. Results expressed as the mean ± SD of 3 individual experiments. In each condition 150 cells were analyzed; *, p<0.05. (**C**) Representative electron micrographs of the cytoplasmic regions of CD4^+^ T cells with productive HIV-1 infection; (**a, b**) autophagosomes (arrows) and budding HIV-1 particles (arrowhead); (**c**) dashed arrows indicate autophagolysosomes with electron-dense structures in HIV-infected CD4^+^ T cells. (**D**) Frequency of autophagosome (**a**) and autophagolysosome (**c**) in HIV-infected CD4^+^ T cells. Budding virus on cell surface was used to monitor infected cells. A total of 150 cells were analyzed. *, p<0.05.
(TIF)

Figure S4 **Inhibition of Beclin 1 and Atg5 reduces autophagy in infected cells.** (**A**) CD4^+^ T cells transfected with either control siRNA (Mock) or siRNAs specifically targeting BECLIN1 and ATG5 were infected with HIV-1. Two sequences for Atg5 were used: sequence 1 (ATG5_1_) and sequence 2 (ATG5_2_). Immunoblots of lysates at day 5 after infection are shown. Membranes were probed for Beclin 1, Atg5 and LC3. Actin was used as a control for protein loading. One representative experiment out to three performed is shown. (**B**) The distribution of LC3-II (number of puncta per cell ≥6) was determined by fluorescence microscopy in Gag^+^ cells. The values shown are means ± SD of three independent experiments (≥200 cells were examined); *, p<0.05.
(TIF)

Figure S5 **HIV-1 infection induces LMP in the absence of Beclin 1 and Atg5.** HIV-infected CD4^+^ T cells were transfected with siRNA specific for BECLIN1 and ATG5 or the control siRNA (mock) and then infected in the absence (NI) or in the presence of HIV-1 (HIV-1). (**A**) At day 5 post-infection, cell extracts were analyzed for Beclin and Atg5. (**B**) Cells were stained with specific antibodies against Cathepsin D (Cat D) and Gag antigen. The subcellular distribution of Cat D in the Gag^+^ cells was analyzed. More than 200 cells were counted for each staining and the results shown are the means ± SD of three independent experiments. No statistical difference was observed. (**C**) Percentage of cell death assessed by flow cytometry using propidium iodide (PI). Results are the means ± SD of three independent experiments. No statistical difference was observed in the absence or presence of specific siRNAs.
(TIF)

Figure S6 **Productive infection induces DRAM.** CD4^+^ T cells were transfected with siRNA specific for p53, DRAM or the control siRNA (mock) and then infected with HIV-1. Cells were then cultured in the absence (bystander) or presence of ConA+IL-2 (activation). (**A**) ΔΨm loss and cell death were assessed using DioC_6_ and propidium iodide (PI), respectively. Flow cytometric analysis shown is performed at day 5 post-infection. A representative experiment is shown and in (**B**), histograms show the means ± SD of three individual experiments. Cells were analyzed on days 4 and 5 post-infection (**C**) Percentage of HIV infection was determined by intracellular staining with specific Gag antibody Gag antigen. (**D**) Western blots of DRAM and LC3 expression in CD4^+^ T cells infected by HIV-1 in the absence or presence of cell activation on day 5 post-infection. Actin was used to a loading control. (**E**) Histograms show the means ± SD of three individual experiments; *, p<0.05.
(TIF)

Figure S7 **Vpr is dispensable for DRAM-mediated LMP and MOMP in HIV-1 infected CD4 ^+^ T cells.** CD4^+^ T cells were transfected with siRNA specific for DRAM or the control siRNA (mock) and then infected with either Wt or Vpr-defective NL4-3 virus (Vpr^−^). (**A**) Immunoblots of lysates from CD4^+^ T cells. Membranes were probed for DRAM and Actin. (**B**) Cells were stained with specific antibodies against cathepsin D (Cat D) (Red), cytochrome C (Cyt c) (yellow) and Gag antigen (green). (**C**) More than 200 cells were counted for each staining shown in **B**, and the results shown are the means ± SD of three independent experiments.
(TIF)

Figure S8 **Nef-mediated LMP is DRAM-independent.** CD4^+^ T cells were transfected with siRNA specific for p53, DRAM or the control siRNA (mock) and then transfected overnight with (**A**) Nef-WT or (**B**) Nef-AS). Cells were stained with specific antibodies against cathepsin D (Cat D) (blue) and cytochrome C (Cyt c) (red). (**C**) ΔΨm loss was assessed using DioC_6_ probe. Histograms show the means ± SD of three individual experiments.
(TIF)
