# Supplementary figures and images for: DRAM Triggers Lysosomal Membrane Permeabilization and Cell Death in CD4+ T Cells Infected with HIV
Source: PLoS Pathog. 2013 May 2;9(5):e1003328. doi: 10.1371/journal.ppat.1003328 (PMC3642063; doi:10.1371/journal.ppat.1003328)

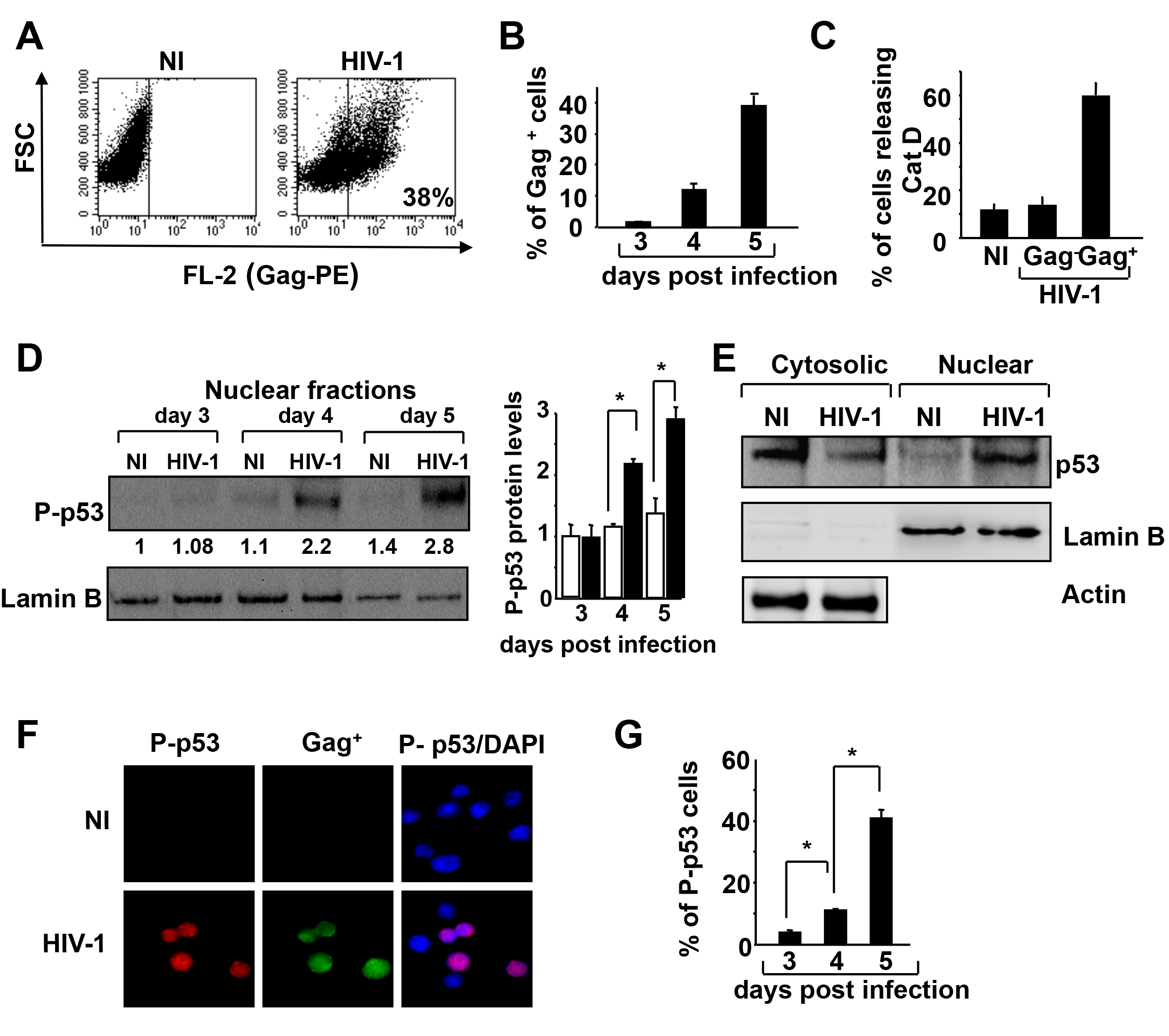

Supplement: Figure S1 — HIV-1 infection induces p53 activation in CD4+ T cells. CD4+ T cells in the absence (NI) or presence of HIV-1LAI (HIV-1) have been analyzed. (A) Flow cytometric analyses of HIV-1 Gag antigen expression (Gag-PE) at day 5 after infection. (B) Percentage of Gag+ cells on days 3, 4 and 5 after infection. Histograms are means ± SD of 10 individual experiments. (C) Percentages of cells releasing cathepsin D (Cat D) in CD4+ T-cells. Cells (Gag+ and Gag-) were analyzed by confocal microscopy, after staining for cathepsin D. Results expressed as the mean ± SD of 10 individual experiments. In each condition 100 cells were counted. (D) Nuclear fractions from CD4+ T cells were analyzed for P-p53 on days 3, 4 and 5 post-infection. Antibody against lamin B was used as a control for protein loading. A typical experiment out of four is shown on the left, and the means ± SD are shown in histograms on the right. *, p<0.05. (E) Nuclear and cytoplasmic fractions from CD4+ T cells were analyzed for p53 on day 5 post-infection. (F) Cells on day 5 were stained with mAbs against P-p53 (red) and p24 antigen (green) and analyzed by fluorescent microscopy. Nuclei were counterstained with DAPI (blue). Representative cells are shown and, in (G), a histogram shows the percentages of cells that are P-p53+ on days 3, 4, and 5. Results expressed as the mean ± SD of 4 individual experiments. In each condition 100 cells were analyzed. *, p<0.05. (TIF) [file ppat.1003328.s001.tif]

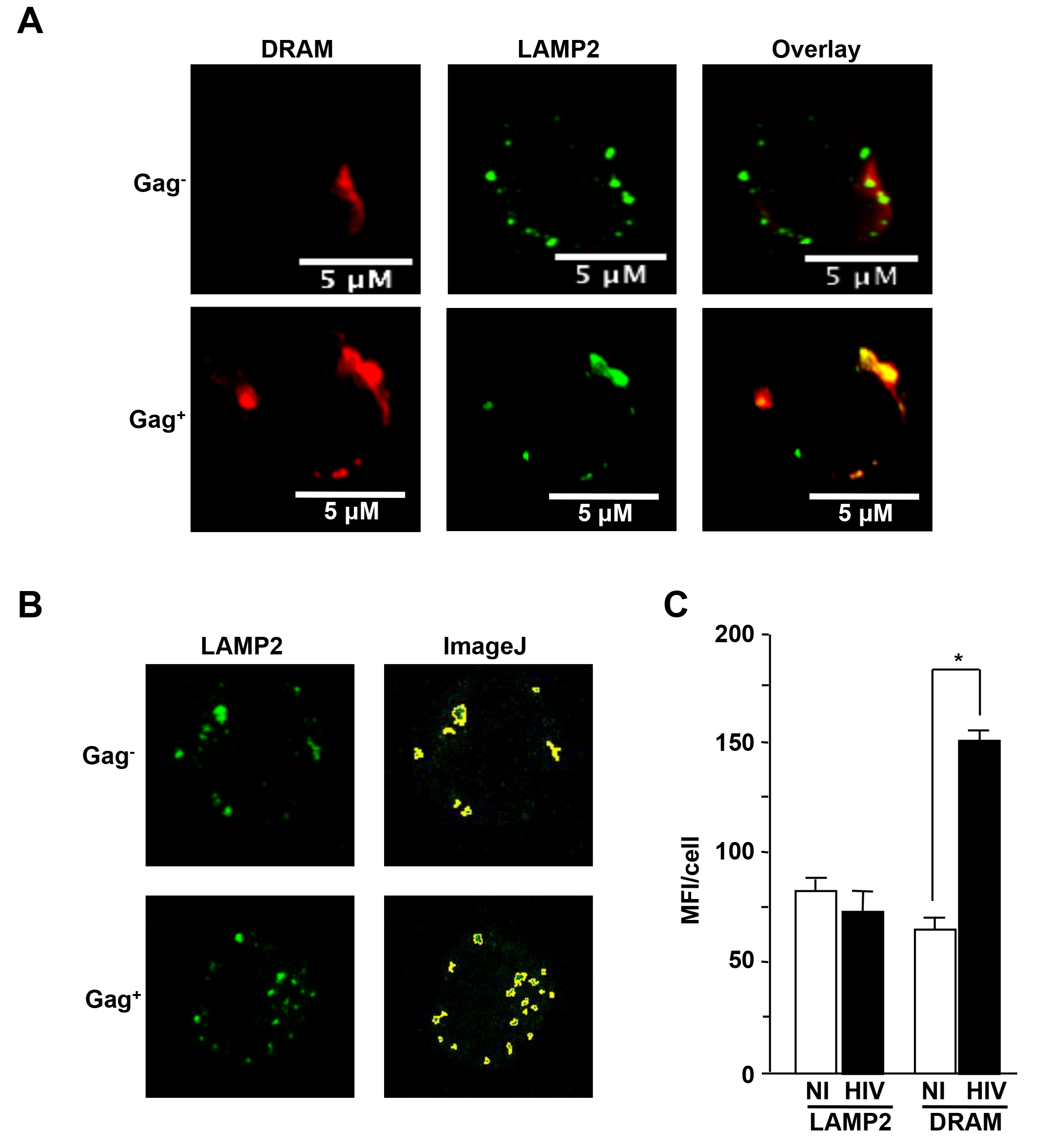

Supplement: Figure S2 — Colocalization of Lamp-2 and DRAM in infected CD4+ T cells. CD4+ T cells are infected with HIV-1 and stained on day 5 post-infection for LAMP2 (green) and DRAM (red). (A) Gag+ and Gag− (NI) cells are shown. (B, C) Quantification of DRAM and LAMP2 expressions was assessed using ImageJ software. For each cell, area and pixel value statistics were calculated accordingly and mean fluorescence intensity per cell is shown. Results expressed as the mean ± SD of 2 individual experiments. In each condition 100 cells were analyzed. *, p<0.05. (TIF) [file ppat.1003328.s002.tif]

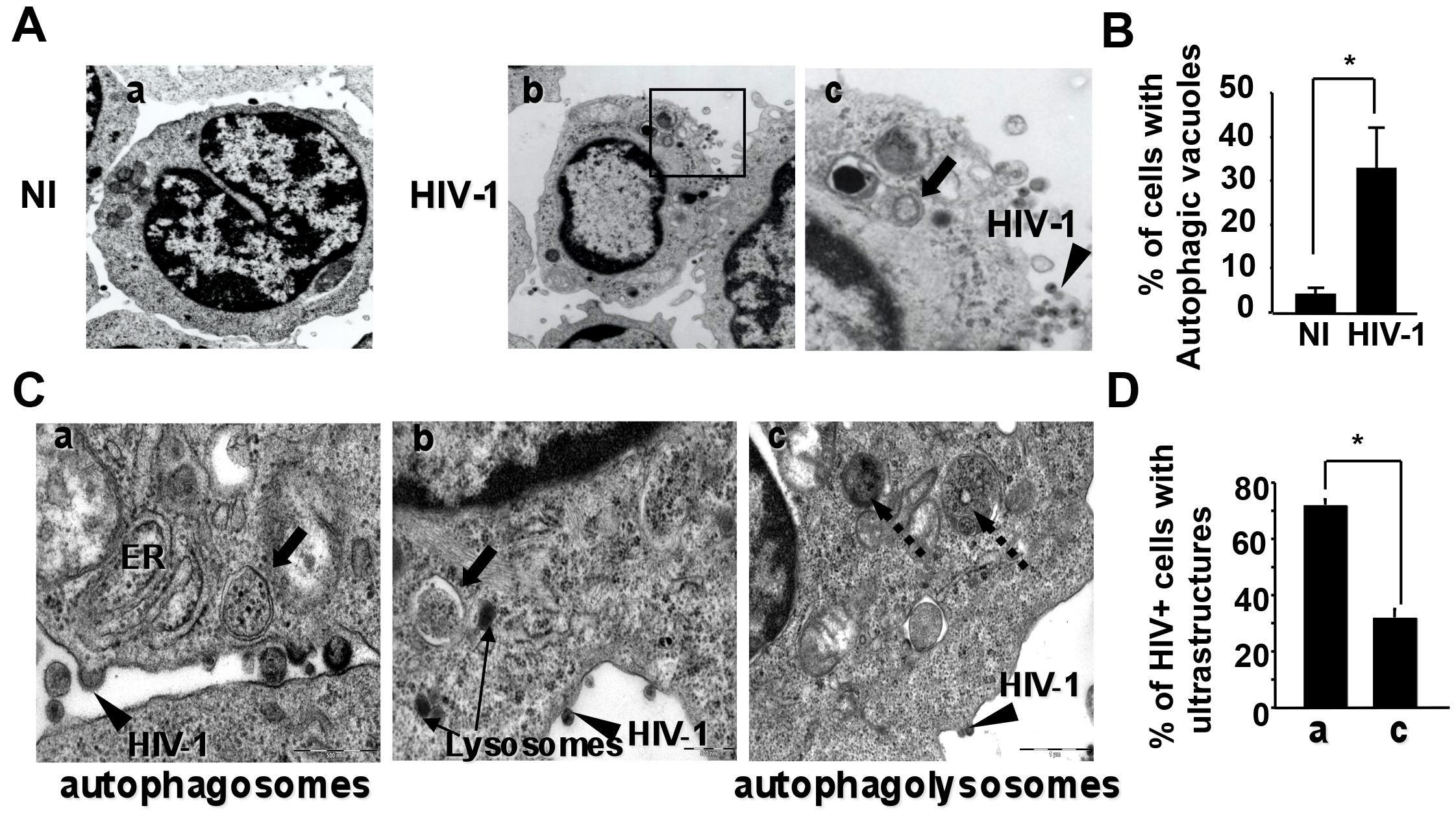

Supplement: Figure S3 — Autophagy-related ultrastructures in CD4+ T infected by HIV. (A) a, b Electron microscopy analyses of autophagy-related ultrastructures in CD4+ T cells in the absence (NI) or presence of HIV-1LAI (HIV-1); (c) higher magnification of the inset in (b); arrows indicate autophagosomes with double-membrane-structures in cells with HIV-1 particles budding at the surface. (B) Quantitation of CD4+ T cells displaying autophagic vacuoles. Results expressed as the mean ± SD of 3 individual experiments. In each condition 150 cells were analyzed; *, p<0.05. (C) Representative electron micrographs of the cytoplasmic regions of CD4+ T cells with productive HIV-1 infection; (a, b) autophagosomes (arrows) and budding HIV-1 particles (arrowhead); (c) dashed arrows indicate autophagolysosomes with electron-dense structures in HIV-infected CD4+ T cells. (D) Frequency of autophagosome (a) and autophagolysosome (c) in HIV-infected CD4+ T cells. Budding virus on cell surface was used to monitor infected cells. A total of 150 cells were analyzed. *, p<0.05. (TIF) [file ppat.1003328.s003.tif]

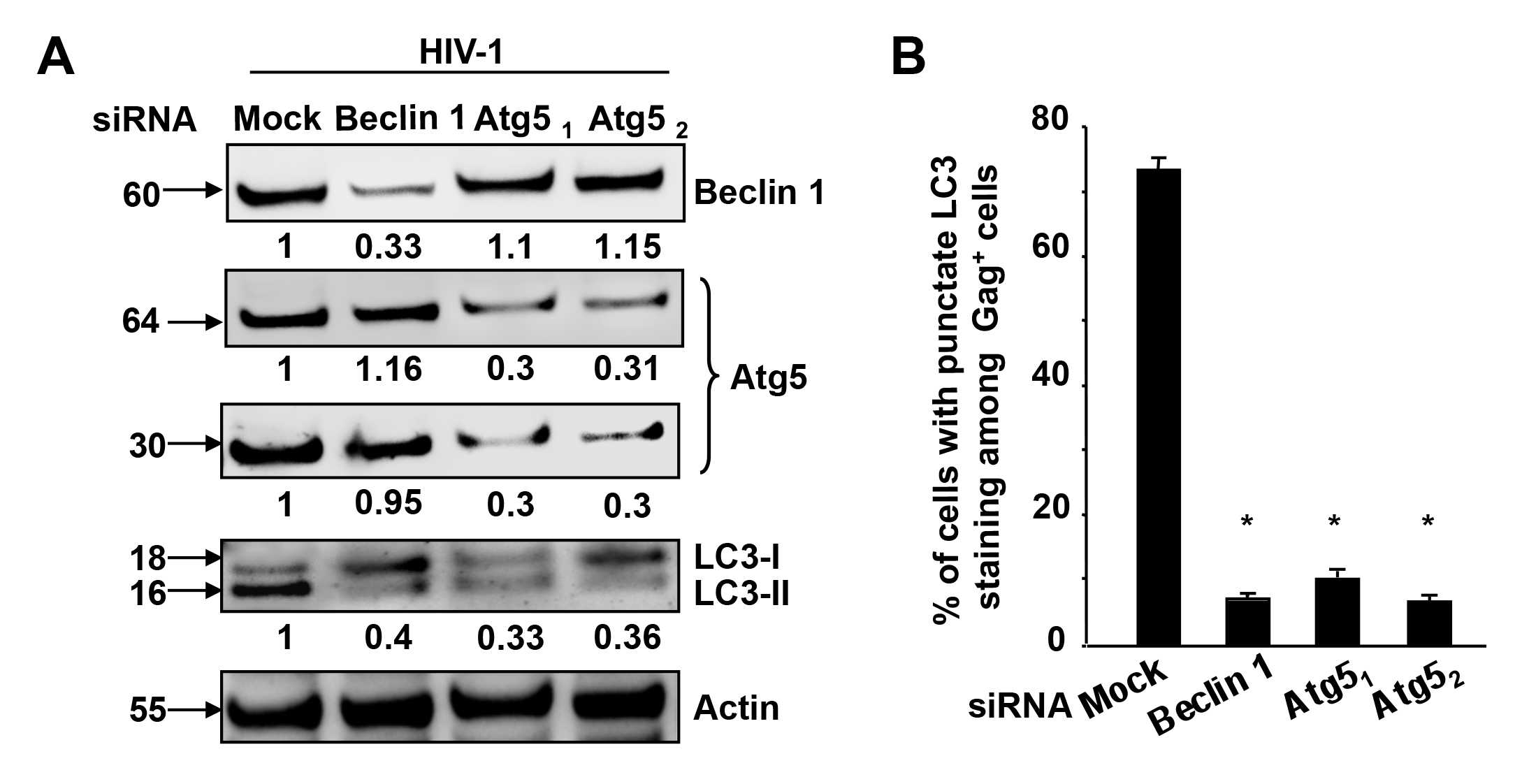

Supplement: Figure S4 — Inhibition of Beclin 1 and Atg5 reduces autophagy in infected cells. (A) CD4+ T cells transfected with either control siRNA (Mock) or siRNAs specifically targeting BECLIN1 and ATG5 were infected with HIV-1. Two sequences for Atg5 were used: sequence 1 (ATG51) and sequence 2 (ATG52). Immunoblots of lysates at day 5 after infection are shown. Membranes were probed for Beclin 1, Atg5 and LC3. Actin was used as a control for protein loading. One representative experiment out to three performed is shown. (B) The distribution of LC3-II (number of puncta per cell ≥6) was determined by fluorescence microscopy in Gag+ cells. The values shown are means ± SD of three independent experiments (≥200 cells were examined); *, p<0.05. (TIF) [file ppat.1003328.s004.tif]

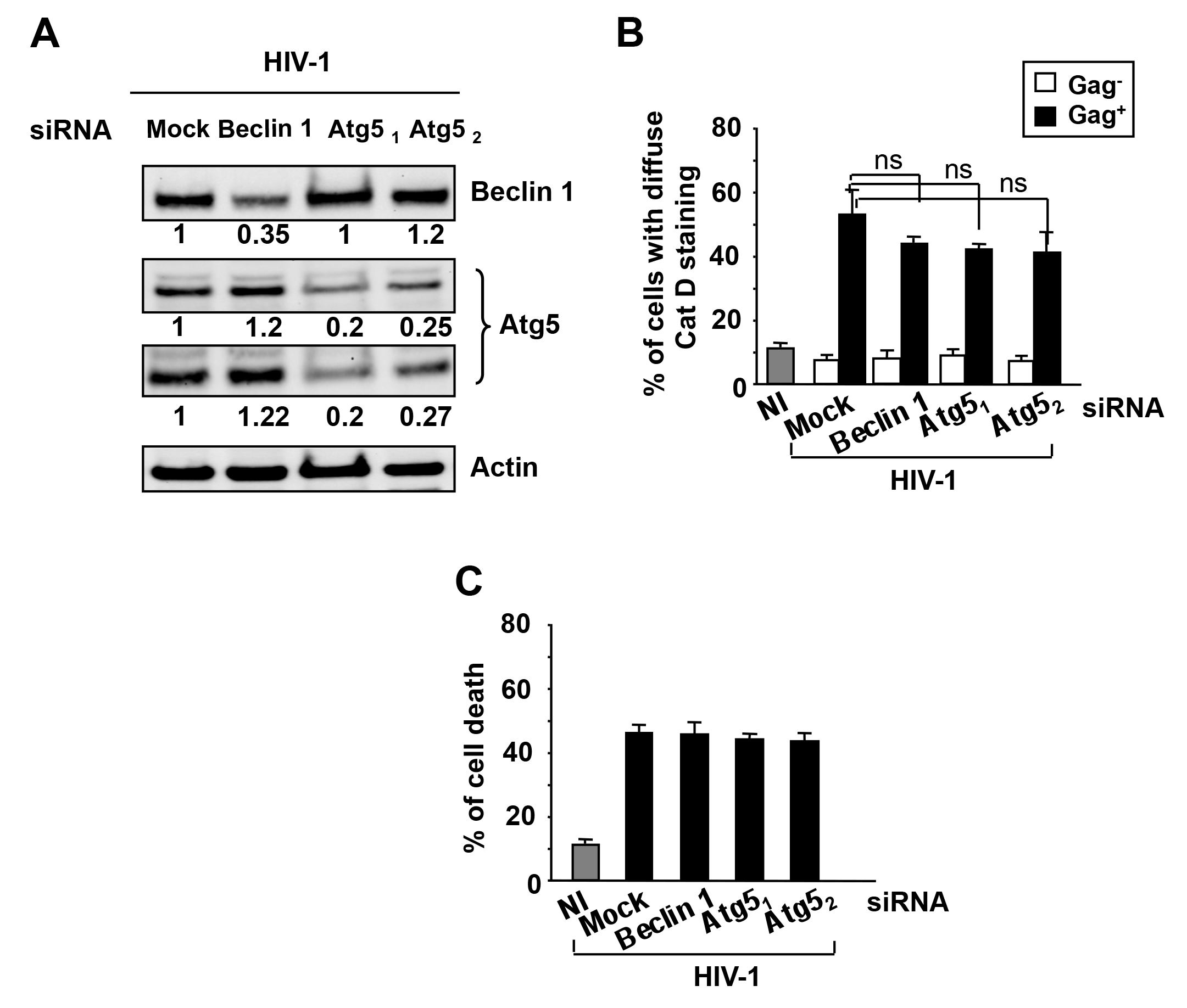

Supplement: Figure S5 — HIV-1 infection induces LMP in the absence of Beclin 1 and Atg5. HIV-infected CD4+ T cells were transfected with siRNA specific for BECLIN1 and ATG5 or the control siRNA (mock) and then infected in the absence (NI) or in the presence of HIV-1 (HIV-1). (A) At day 5 post-infection, cell extracts were analyzed for Beclin and Atg5. (B) Cells were stained with specific antibodies against Cathepsin D (Cat D) and Gag antigen. The subcellular distribution of Cat D in the Gag+ cells was analyzed. More than 200 cells were counted for each staining and the results shown are the means ± SD of three independent experiments. No statistical difference was observed. (C) Percentage of cell death assessed by flow cytometry using propidium iodide (PI). Results are the means ± SD of three independent experiments. No statistical difference was observed in the absence or presence of specific siRNAs. (TIF) [file ppat.1003328.s005.tif]

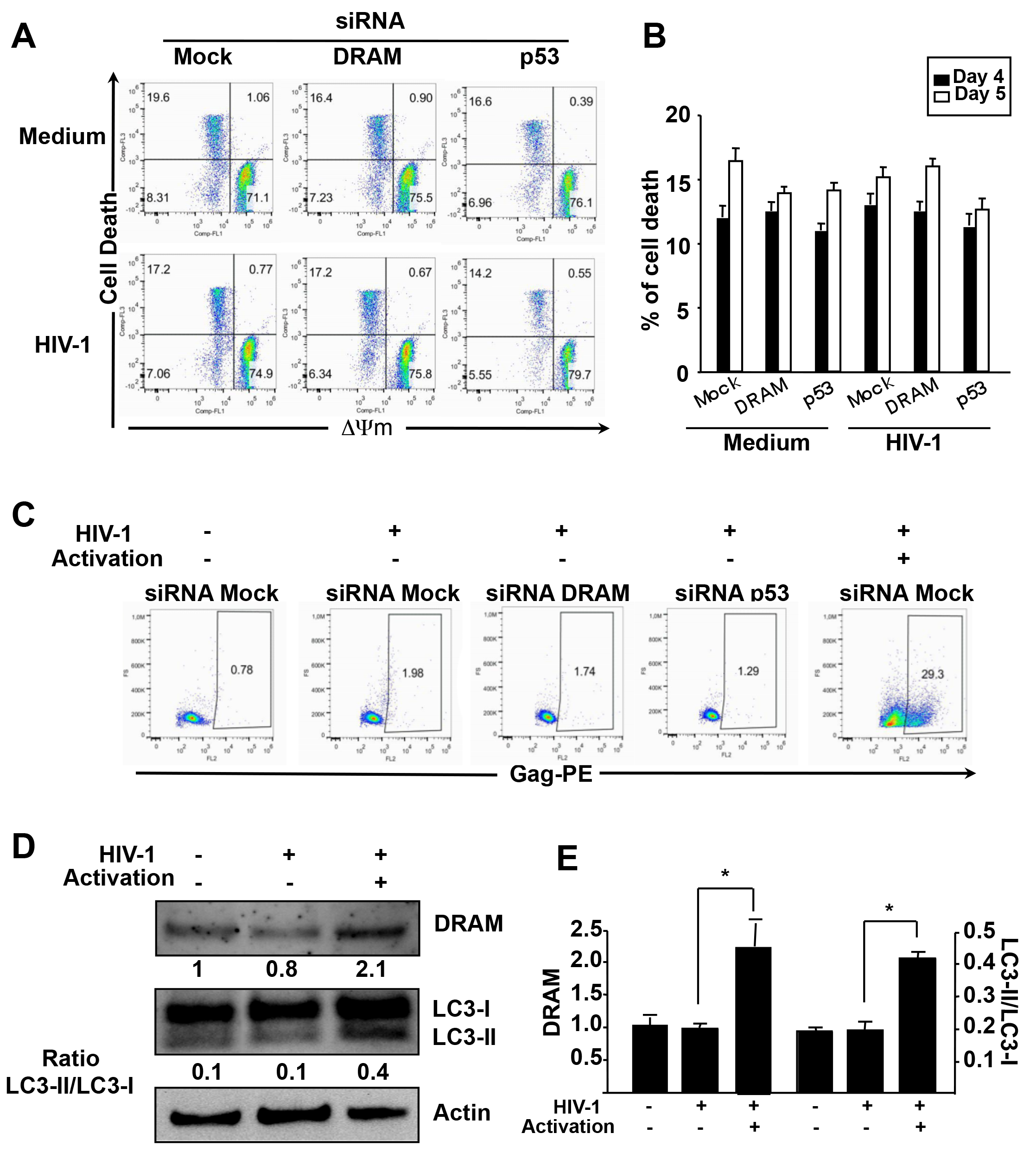

Supplement: Figure S6 — Productive infection induces DRAM. CD4+ T cells were transfected with siRNA specific for p53, DRAM or the control siRNA (mock) and then infected with HIV-1. Cells were then cultured in the absence (bystander) or presence of ConA+IL-2 (activation). (A) ΔΨm loss and cell death were assessed using DioC6 and propidium iodide (PI), respectively. Flow cytometric analysis shown is performed at day 5 post-infection. A representative experiment is shown and in (B), histograms show the means ± SD of three individual experiments. Cells were analyzed on days 4 and 5 post-infection (C) Percentage of HIV infection was determined by intracellular staining with specific Gag antibody Gag antigen. (D) Western blots of DRAM and LC3 expression in CD4+ T cells infected by HIV-1 in the absence or presence of cell activation on day 5 post-infection. Actin was used to a loading control. (E) Histograms show the means ± SD of three individual experiments; *, p<0.05. (TIF) [file ppat.1003328.s006.tif]

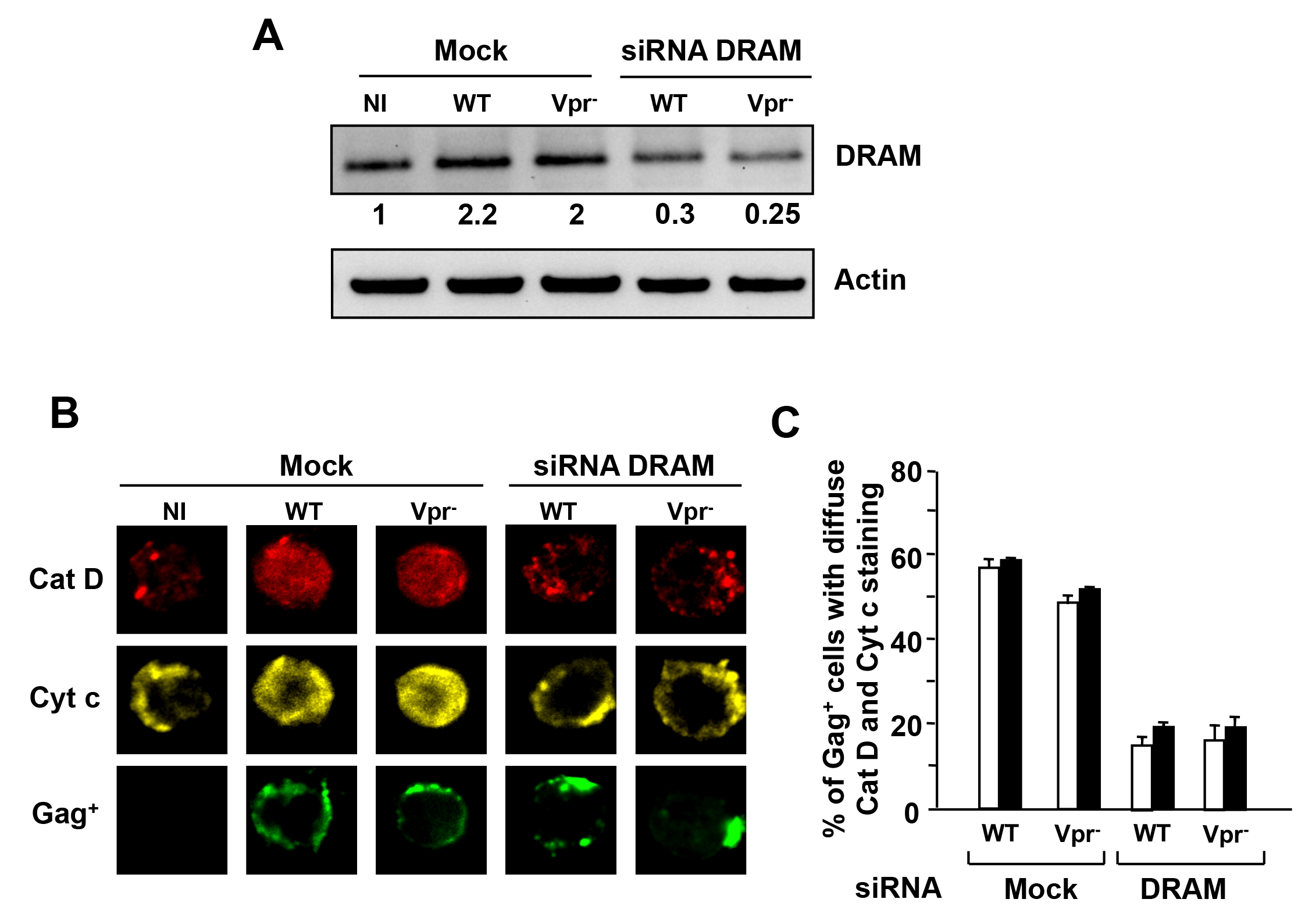

Supplement: Figure S7 — Vpr is dispensable for DRAM-mediated LMP and MOMP in HIV-1 infected CD4 + T cells. CD4+ T cells were transfected with siRNA specific for DRAM or the control siRNA (mock) and then infected with either Wt or Vpr-defective NL4-3 virus (Vpr−). (A) Immunoblots of lysates from CD4+ T cells. Membranes were probed for DRAM and Actin. (B) Cells were stained with specific antibodies against cathepsin D (Cat D) (Red), cytochrome C (Cyt c) (yellow) and Gag antigen (green). (C) More than 200 cells were counted for each staining shown in B, and the results shown are the means ± SD of three independent experiments. (TIF) [file ppat.1003328.s007.tif]

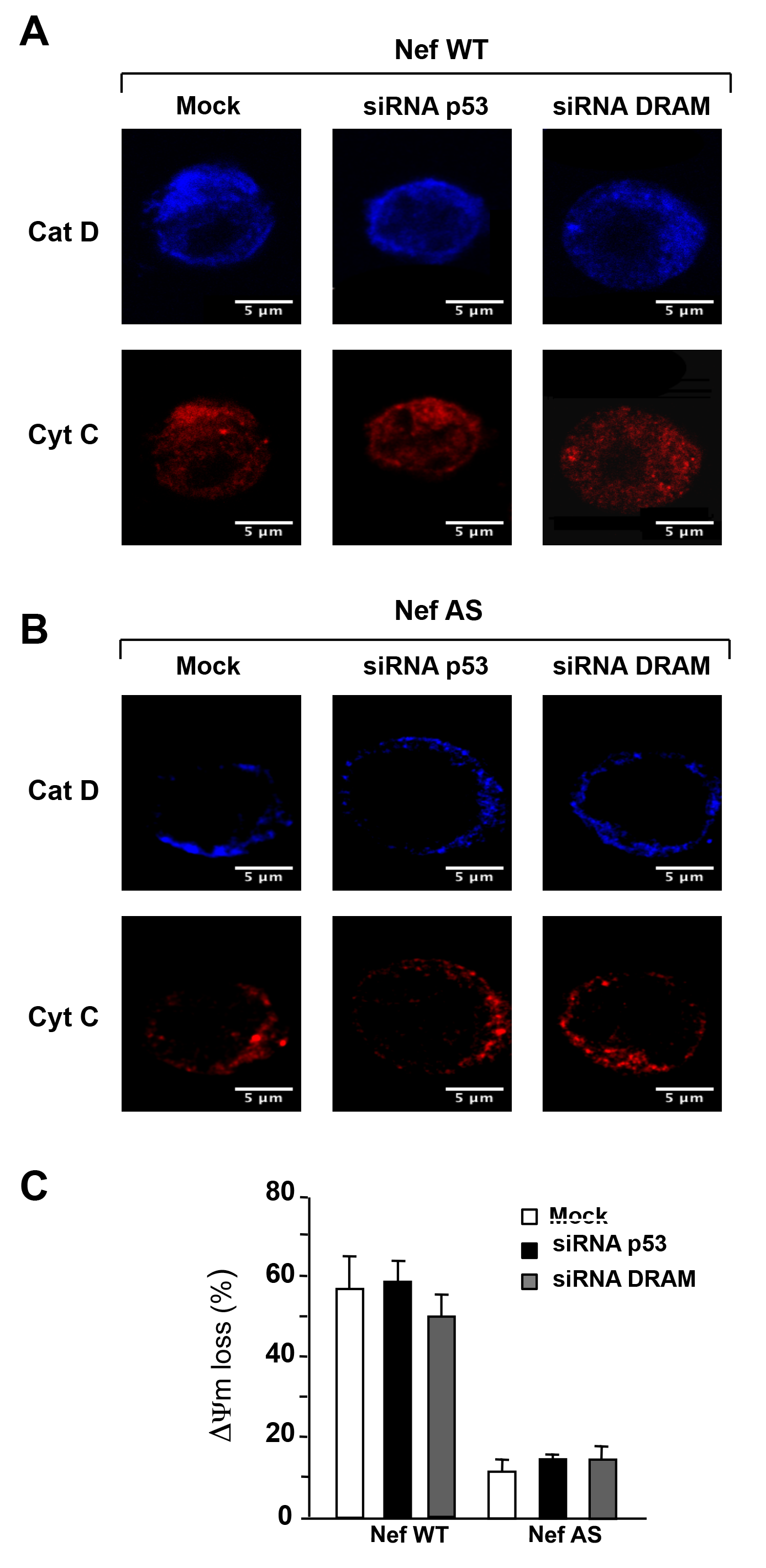

Supplement: Figure S8 — Nef-mediated LMP is DRAM-independent. CD4+ T cells were transfected with siRNA specific for p53, DRAM or the control siRNA (mock) and then transfected overnight with (A) Nef-WT or (B) Nef-AS). Cells were stained with specific antibodies against cathepsin D (Cat D) (blue) and cytochrome C (Cyt c) (red). (C) ΔΨm loss was assessed using DioC6 probe. Histograms show the means ± SD of three individual experiments. (TIF) [file ppat.1003328.s008.tif]
